# Notice to Exchanges

**Published:** 1905-12

**Authors:** 


### NOTICE TO EXCHANGES.

This, the December number of the International Dental Journal, will be the last issue. Exchanges will kindly take note and strike this periodical from their list.

